# A Treatise on the Nervous Diseases of Children, for Physicians and Students

**Published:** 1906-09

**Authors:** 


**A Treatise on the Nervous Diseases of Children, for Physicians and Students.** By **B. Sachs**, M.D., Alienist and Neurologist to Bellevue Hospital; Neurologist to the Mt. Sinai Hospital, consulting physician to Manhattan State Hospital, east and west. Second edition, revised. Octavo. pp. 12-571. New York: William Wood & Co. (Price: $4.25).

This work being written by a trained neurologist of wide experience, it is interesting to note the position Sachs takes in regard to certain mooted questions. In his chapter on epilepsy, he says of eyestrain, of urethral stricture, of narrow prepuces and of laryngeal irritation: "I doubt if any one of these conditions has ever been the sole cause of epilepsy, though I am willing to conceed that they may be sufficient to produce occasional attacks in persons with this special hereditary taint." In view of the strong anti-bromide position taken by Dr. Spratling, of Sonyea, it is important to observe that Sachs says, "up to this time, we have found no drugs that can in any sense be considered proper substitutes for the bromides." He is outspoken in utterly condemning the cutting of the eye muscles in epilepsy. In the treatment of chorea, he places rest in bed in the foreground, milk diet coming next in importance. In using arsenic, he is far more cautious than was Seguin. Sachs would rather abandon the use of arsenic than push it to the extreme that Seguin did. We do not agree with the author in his discussion of headache and eye strain. He is "firmly convinced that eye strain is the sole cause of headache in relatively few instances, and that these are located over the eyebrows." In our experience the location of the headache dut to eye strain is quite as often occipital in location.

The description of the diseases of the spinal cord is most admirable, while the work as a whole is an excellent presentation of the subject as it is taught today. The author's personality is strongly felt throughout the book. We feel that this work fills a distinct gap in medical literature. J. W. P.

